# Energy Values and Standardized Ileal Digestibility of Amino Acids in Fermented Soybean Meal Fed to Growing Pigs

**DOI:** 10.3390/ani14202945

**Published:** 2024-10-12

**Authors:** Jinxiu Huang, Ya Wang, Zhiyun Liu, Ruyue Ma, Xiaoxia Zhong, Yanchu Yao

**Affiliations:** Institute of Animal Nutrition, Chongqing Academy of Animal Sciences, Chongqing 402460, China; short00@163.com (J.H.); wangya920708@163.com (Y.W.); liuzhiyun2009.6@163.com (Z.L.); 13883137707@163.cm (R.M.); zhongchuck@163.com (X.Z.)

**Keywords:** growing pigs, energy, amino acid digestibility, fermented soybean meal

## Abstract

**Simple Summary:**

Fermentation not only reduces the anti-nutritional factors in soybean meal, but also increases the contents of bioactive peptides and free amino acids, and promotes the digestibility of nutrients in soybean meal for growing pigs. The enzymes and prebiotics contained in fermented soybean meal (FSBM) improve the intestinal health, antioxidant capacity, and immune function of animals. However, there is a lack of systematic evaluation of the nutritional value of FSBM for growing pigs. Therefore, we selected 10 representative FSBM samples from nine different manufacturers in China, and determined the digestible energy (DE), metabolizable energy (ME), and standardized ileal digestibility (SID) of amino acids (AA) of FSBM. We found that the mean values of DE, ME, and SID of AA in the FSBM samples were similar to those of NRC (2012), but the variation was large. The DE and ME values can be predicted based on the chemical composition of FSBM in growing pigs.

**Abstract:**

This study aimed to evaluate the digestible energy (DE), metabolizable energy (ME), and standardized ileal digestibility (SID) of amino acids (AA) in fermented soybean meal (FSBM) fed to growing pigs. In experiment 1, twenty-two growing pigs (Duroc × Landrace × Yorkshire; 32.0 ± 4.2 kg BW) were fed one of 11 diets in a replicated 11 × 3 incomplete Latin square design to determine the DE and ME of FSBM. The diets included a corn-based diet and 10 experimental diets formulated by replacing the corn with 29.10% FSBM. In experiment 2, eleven growing pigs (Duroc × Landrace × Yorkshire; 24.3 ± 2.3 kg BW) were surgically equipped with a T-cannula and arranged in an 11 × 6 incomplete Latin square design with 11 diets and six periods. The diets included an N-free diet and 10 experimental diets containing 40% FSBM as the sole source of AA. The results showed that the contents of dry matter (DM), gross energy, crude protein, crude fiber, neutral detergent fiber, acid detergent fiber, calcium, and phosphorus averaged 91.83% (ranging from 89.24 to 94.55%), 18.44 MJ/kg (ranging from 18.00 to 18.95 MJ/kg), 50.63% (ranging from 50.00 to 51.81%), 4.51% (ranging from 3.41 to 5.40%), 9.17% (ranging from 7.02 to 10.30%), 6.38% (ranging from 4.97 to 7.45%), 0.32% (ranging from 0.29 to 0.34%), and 0.67% (ranging from 0.61 to 0.84%), respectively. The average DE and ME were 17.55 and 16.33 MJ/kg DM, respectively, with ranges of 15.72 to 18.80 MJ/kg DM and 14.30 to 17.72 MJ/kg DM, respectively. Significant differences were observed in the SID of essential AA (*p* < 0.05), except for Thr and Arg. The SID of Lys, Met, Trp, and Thr ranged from 68.13 to 83.27% (mean = 76.62%), 62.23 to 89.30% (mean = 75.25%), 72.30 to 90.29% (mean = 80.96%), and 74.17 to 84.41% (mean = 77.90%), respectively. These results indicate significant variability in chemical composition, energy content, and the SID of AA among the selected FSBM samples. The chemical composition of FSBM can be used to predict DE and ME values.

## 1. Introduction

Soybean meal is a byproduct obtained after soybean oil extraction, containing a high crude protein (CP) content (43%) with a relatively balanced amino acid (AA) composition, and serves as an important plant protein source for swine (NRC, 2012) [1]. However, soybean meal contains a variety of anti-nutritional factors, such as antigenic proteins, urease, and trypsin inhibitors (Woyengo et al., 2017) [2], which reduce nutrient digestibility by reducing digestive enzyme activity and impairing intestinal health (Zentek and Boroojeni, 2020) [3], especially for young animals with an immature digestive system. This may lead to health issues, thereby limiting their application in animal diets (Zhang et al., 2013) [4].

Through biological fermentation technology, the CP content of soybean meal can be increased (Chen et al., 2010) [5], and the anti-nutritional factors in soybean meal, such as urease and trypsin inhibitors, can be reduced (Jeong et al., 2016) [6]. The soybean proteins are degraded into small peptides and free AA, which not only improve the quality and nutritional value of soybean meal but also enhance feed utilization (Chen et al., 2010) [5]. Li et al. (2019) found that protein digestibility increased from 57.99% to 95.96% when using fermented soybean meal (FSBM) [7]. FSBM also contains a rich variety of enzymes and probiotics, which assist in animal digestion and promote intestinal health. FSBM increases nutrient digestibility by improving the intestinal function, anti-oxidative capacity, and immune function of weaned pigs (Yan et al., 2022) [8].

The quality of FSBM produced in the market is uneven due to differences in selected soybean meal, strain used, and fermentation technology. Currently, the nutritional value database for FSBM in growing pigs is incomplete. Therefore, this study collected ten representative FSBM samples from nine different manufacturers in China to evaluate their nutritional value as well as to generate prediction equations for DE and ME based on chemical analysis in growing pigs. This provides a reference for the precise application of FSBM in the diets of growing pigs.

## 2. Materials and Methods

The experimental protocols, including animal care and use, were reviewed and approved by the Animal Care and Use Committee at the Chongqing Academy of Animal Sciences (XKY-20221203).

### 2.1. Fermented Soybean Meal Collection

**Figure 1:**
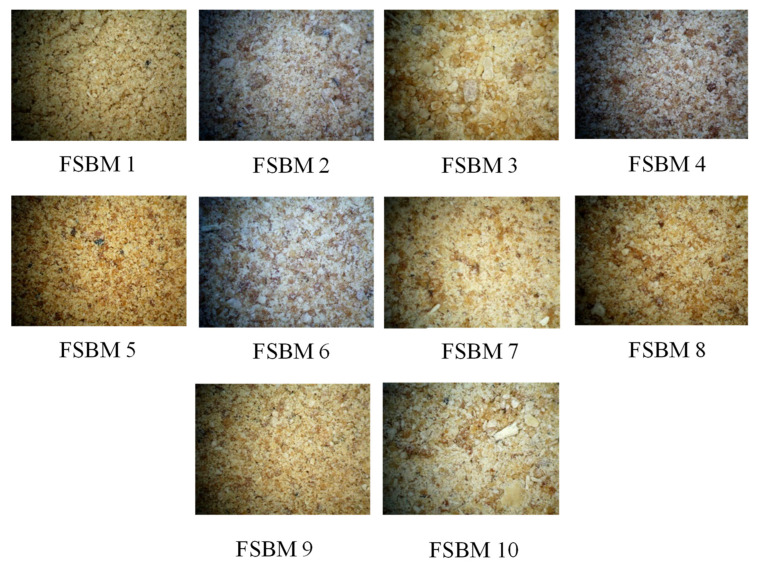
Microscope photos of fermented soybean meal (FSBM) samples.

We selected 10 representative samples of FSBM from 9 different manufacturers in China. The information for these 10 FSBM samples is shown in Table 1. Microscope photos of FSBM samples were shown in Figure 1. The chemical composition, AA composition, and mineral and anti-nutrient factor content of FSBM samples were shown in Table 2.

### 2.2. Experiment 1: Available Energy Measurements

#### 2.2.1. Animals, Diets, and Experimental Design

Experiment 1 was conducted to determine the DE and ME in 10 FSBM samples for the growing pigs. Twenty-two growing male pigs (Duroc × Landrace × Yorkshire; initial body weight (BW) of 32.0 ± 4.2 kg) were assigned to a replicated 11 × 3 incomplete Latin square design, including a corn-based diet and 10 FSBM diets and three consecutive periods. Each period had a 7-day adaption and 5-day total feces and urine collection. The pigs and periods were assigned according to the blocking criteria. A corn-based diet was formulated with the corn as the single energy source, and for the FSBM diets, FSBM accounted for 29.10% of the diets, with FSBM replacing partial corn in the corn-based diet (Table 3). Vitamins and minerals were added to meet or exceed the requirements recommended by the NRC (2012) [1]. The analyzed composition of diets in experiment 1 are presented in Table 4. The pigs were individually housed in the metabolism crates (1.8 × 0.7 × 0.9 m) and stayed in a temperature controlled room (22 ± 2 °C). Each cage was equipped with a feeder, a nipple drinker, and a fully slatted plastic floor, and there was a colleting plate under each cage. All pigs had ad libitum access to drinking water, and the feces and urine could be collected completely and separately. Each pig was weighed at the beginning of each period and fed at a daily level of 4% of their initial BW (Adeola, 2001) [9]. Daily diets were divided into two equal-sized meals and fed to the growing pigs at 0800 and 1500 h, respectively.

#### 2.2.2. Sample Collection and Preparation

During the total collection period, the feed intake, fecal output, and total urine volume of each pig were accurately recorded every day. Urine was collected using the time-to-time method as described by Pedersen et al. (2007) [10]. In each collection period, a plastic bucket containing 50 mL of 6 *N* HCl was used to collect the urine from the drainage tube of the collecting basin under the metabolism crate, and the total urine volume for each pig was recorded. The 10% of the total urine subsample from each pig was collected and stored at −20 °C. A total of 4 mL of urine was dried with filter paper in an air-forced oven at 65 °C for 8 h to determine the GE (Lyu et al., 2022) [11]. Feces were collected immediately when they appeared in the metabolism cage, and then transferred into the plastic bags and stored at −20 °C. At the end of each period, the feces collected on different collection day were thawed, pooled, homogenized, and weighed. Then, about 500 g of fecal subsample was taken and dried in an air-forced oven at 65 °C for 72 h.

### 2.3. Experiment 2: Determination of Amino Acid Digestibility

#### 2.3.1. Animals, Diets, and Experimental Design

Experiment 2 was conducted to determine the AID and SID of AA in 10 FSBM samples for the growing pigs. Eleven growing male pigs (Duroc × Landrace × Yorkshire; initial BW = 24.3 ± 2.3 kg) equipped with a T-cannula in the distal ileum were allotted to an 11 × 6 incomplete Latin square design with 11 diets and 6 consecutive periods. The pigs and periods were considered as the blocking factors, and there were six replicate pigs per treatment. The 11 dietary treatments included a nitrogen-free diet and 10 selected FSBM diets. The FSBM diets were formulated with the selected FSBM as the only source of CP and AA. A nitrogen-free diet was used to determine the basal endogenous losses of AA. The ingredients and AA composition of these experimental diets were shown in Table 5 and Table 6. Vitamins and minerals were supplemented to meet or exceed the nutrient requirement for 20 to 50 kg growing pigs recommended by the NRC (2012) [1]. All diets in experiment 2 contained 0.20% of titanium dioxide (TiO_2_) as an indigestible marker for calculating AA digestibility. Pigs were individually housed in metabolism crates (1.8 × 0.7 × 0.9 m) and stayed in an environmentally controlled room (22 ± 2 °C). Each crate was equipped with a nipple drinker and a feeder. All pigs had ad libitum access to drinking water. Each pig was weighed at beginning of each period and fed at a daily level of 4% of their initial BW (Adeola, 2001) [9]. Daily diets were divided into two equal-sized meals and fed to the growing pigs at 0800 and 1700 h, respectively. Each period consisted of a 5-day adaptation followed by 2-day ileal digesta collection.

#### 2.3.2. Sample Collection and Preparation

In each period, ileal digesta samples were collected continuously for 12 h from 08:00 to 20:00 h on days 6 and 7 as described by Stein et al. (2007) [12]. At the end of each period, all of the ileal digesta samples were thawed and pooled for each pig. Then, the digesta subsamples were freeze-dried for the AA analysis.

### 2.4. Chemical Analysis

In experiment 1, the FSBM, experimental diets, and dried feces samples were analyzed for different indices using the national standard methods (announced by the General Administration of Quality Supervision, Inspection and Quarantine of the People’s Republic of China and Standardization Administration of China) unless otherwise stated. The percentages of dry matter (DM; GB/T 6435-2014) [13], CP (GB/T 6432-2018) [14], ash (GB/T 6438-2007) [15], ether extract (EE; GB/T 6433-2006) [16], starch (GB/T 20194-2018) [17], calcium (Ca, GB/T 6436-2018) [18], phosphorus (P, GB/T 6437-2018) [19], and other minerals (potassium (K), sodium (Na), magnesium (Mg), copper (Cu), iron (Fe), manganese (Mn), and zinc (Zn)) (GB/T 13885-2017) [20] were determined in the aforementioned samples. The gross energy (GE) of the FSBM, experimental diets, feces, and urine were determined using an oxygen bomb automatic calorimeter (Model Parr 6400, Parr Instrument Company, Moline, IL, USA). Crude fiber (CF), neutral detergent fiber (NDF), and acid detergent fiber (ADF) values were determined using an automatic fiber analyzer (A2000I; Ankom Technology, Macedon, NY, USA) (Li et al., 2015) [21]. The determination of trypsin inhibitor content, soy globulins, and β-conglycinin was conducted using the HPLC-MS/MS system employed in the study, which comprised an Agi-lent 1200 HPLC unit and an Agilent 6460 000 Triple Quad mass spectrometer.

In experiment 2, the FSBM diets and ileal digesta were analyzed for amino acids using an amino acid analyzer (Model L-8900, Hitachi, Tokyo, Japan). The tryptophan was determined by a high-performance liquid chromatograph (HPLC; WATERS e2695 Series; Waters, Santa Clara, CA, USA). The concentration of titanium dioxide in the diets and ileal digesta was determined using an ICP-AES according to the method of GB/T 5009.246-2016 [22].

### 2.5. Calculations

In experiment 1, the DE and ME of the FSBM diets were calculated as described by Adeola (2001) [9] as follows:

DE_d_ (MJ/kg DM) = (GE_i_ − GE_f_)/F

DE_dc_ (MJ/kg DM) = DE_d_/0.97

DE_FSBM_ (MJ/kg DM) = [ DE_d_ − DE_dc_×(100% − 29.1%)]/29.1%

ME_d_ (MJ/kg DM) = (GE_i_ − GE_f_ − GE_u_)/F

ME_dc_ (MJ/kg DM) = ME_d_/0.97

ME_FSBM_ (MJ/kg DM) = [ME_d_ − ME_dc_ × (100% − 29.1%)]/29.1%

where DE_d_ and ME_d_ are the DE and ME values in each diet; GE_i_ is the total GE intake of each pig calculated as the product of the GE content of the diet over F, which was the actual feed intake over the 5d collection period; GE_f_ and GE_u_ are the GE content in the feces and urine of each pig over the 5d collection period; DE_dc_ and ME_dc_ are the adjusted DE and ME in the basal diet and 0.97 is the percentage of the ingredients that supplied energy in the diet; DE_FSBM_ and ME_FSBM_ are the DE and ME values in each FSBM sample; and 29.1% is the percentage of energy supplied by FSBM in the basal diet.

In experiment 2, the apparent ileal digestibility (AID), standardized ileal digestibility (SID), and ileal endogenous losses of AA (IAA) were calculated as described by Stein et al. (2007) [12] using the following equation:

AID, % = [1 − (AA_1_/AA_2_) × (IM_2_/IM_1_)] × 100;

where IM_1_ and IM_2_ are titanium dioxide content (mg/kg DM) in digesta and diet; AA_1_ and AA_2_ are AA content (mg/kg DM) in digesta and diet.

IAA was calculated from the growing pigs fed the N-free diet as follows:

IAA = AA_3_ × (IM_4_/IM_3_);

where IAA is the basal ileal endogenous loss of AA (mg/kg DM intake). AA_3_ and IM_3_ are AA and titanium dioxide content (mg/kg DM) in the ileal digesta fed the N-free diet; IM_4_ is the titanium dioxide content (mg/kg DM) in the N-free diet.

SID, % = AID + (IAA/AA_2_) × 100.

### 2.6. Statistical Analyses

Data in experiment 1 and experiment 2 were analyzed using SAS 9.2 software (SAS Inst. Inc., Carry, NC, USA). At first, the normality tests were performed using the UNIVARIATE procedure of SAS with Normal and PLOT options, and then Levene’s tests was used to evaluate the heterogeneity of variances. When the data met the requirements of the normality and variance homogeneity in different treatments, they were then analyzed using the PROC MIXED procedure. The statistical model included the fixed effects of FSBM and the random effects of pig and period. The Tukey test was used for multiple population comparison among different treatments with letter grouping. For all statistical analyses, significance and tendency were declared at *p* < 0.05 and 0.05 ≤ *p* < 0.10, respectively.

The relationship between energy content and chemical composition in experiment 1 was analyzed using the PROC CORR procedure in SAS. Using the stepwise regression procedure (PROC REG) in SAS (Kaps and Lamberson, 2017) [23], we developed DE and ME prediction equations for growing pigs. The R^2^, *p*-value, root mean square error (RMSE) and Akaike’s information criterion (AIC) were used to identify the best-fit equations. Equations with the greatest R^2^ and the least RMSE were proposed to be the best fit.

## 3. Results

### 3.1. Chemical Composition of FSBM Samples

As shown in Table 2, the coefficient of variation (CV) of DM, GE, CP, ash, and nitrogen-free extract (NFE) contents was no more than 5.57%, while the CV of EE, CF, NDF, ADF, starch, and antinutritional factors (phytic acid, glycinin, β-conglycinin, trypsin inhibitor, and lectin) was greater than 12%. The averaged contents of DM, CP, ash, and NFE in the 10 FSBM samples were 91.83%, 50.63%, 6.27%, and 86.14%, respectively. The averaged contents of EE, CF, NDF, ADF, and starch in the 10 FSBM samples were 1.16% (0.69 to 1.75%), 4.51% (3.41 to 5.40%), 9.17% (7.02 to 10.30%), 6.38% (4.97 to 7.45%), and 1.38% (0.49 to 2.50%), respectively. AA contents were relatively stable with CV < 10% (except Thr, CV = 11.71%), the averaged contents of Lys, Met, Trp, and Thr in the 10 FSBM samples were 2.96%, 0.55%, 0.66%, and 2.05%, respectively. There was considerable variation in mineral contents (Table 7); the averaged contents of P, Zn, Fe, Mn, and Na in the 10 FSBM samples were 0.67%, 49.20 mg/kg, 186.07 mg/kg, 36.55 mg/kg, and 212.55 mg/kg, respectively, with the CV > 10%. The averaged contents of Ca, Cu, Mg, and K in the 10 FSBM samples were 0.32%, 12.70 mg/kg, 0.35, and 24.50 g/kg, respectively, with the CV < 10%. The contents of anti-nutritional factors are presented in Table 8; the averaged contents of phytic acid, glycinin, β-conglycinin, trypsin inhibitor, and lectin in the 10 FSBM samples were 0.25%, 57.74 mg/g, 21.28 mg/g, 7.90 mg/g, and 4.45 mg/g, respectively, with the CV > 10%.

### 3.2. Available Energy in FSBM

As shown in Table 9, the contents of DE, ME, and the ratio of ME:DE in the basal diet was 16.12 MJ/kg DM, 15.72 MJ/kg DM, and 97.54%, respectively. The averaged contents of DE, ME, and the ratio of ME:DE in 10 FSBM diets were 15.92 and 15.32 MJ/kg DM, respectively, with ranges of 15.60 to 16.36 MJ/kg DM, 14.96 to 15.81 MJ/kg DM, and 95.42 to 96.82%, respectively.

As shown in Table 10, on a DM basis, there was a significant difference or statistically different trend in the DE and ME contents, and their ratio (*p* ≤ 0.07). The contents of DE, ME, and the ratio of ME:DE ranged from 15.72 to 18.80 MJ/kg, 14.30 to 18.05 MJ/kg, and 89.24% to 96.03%, respectively. The ME content in FSBM sample 3 was greater than that in samples 2, 4, 5, and 9 (*p* = 0.03), whereas it was not different from that in the others. The ratio of ME:DE in FSBM 3 was also greater than that in FSBM 2, 8, 5, 9, and 1 (*p* = 0.01), and was not different from FSBM 6, 7, 10, and 4. FSBM 6, 7, and 10 had greater ratios of ME:DE than FSBM 5, 9, and 1, and the ratio of ME:DE in FSBM 4 was greater than that in FSBM 1 (*p* = 0.01).

### 3.3. Correlation and Prediction Equations for DE and ME

As shown in Table 11, the DM was significant positively correlated with GE contents (r = 0.901; *p* < 0.01). The EE was significant negatively correlated with PA contents (r = −0.654; *p* < 0.05). The CF was significantly positively correlated with NDF (r = 0.785; *p* < 0.01) and ADF (r = 0.947; *p* < 0.01), but the CF had a differential tendency to be negatively correlated with DE (r = −0.785; *p* < 0.10) and ME (r = −0.947; *p* < 0.10). The NDF was significantly positively correlated with ADF contents (r = 0.854; *p* < 0.01). Ash was significant positively correlated with starch content (r = 0.755; *p* < 0.05). The GE had a differential tendency to be negatively correlated with PA (r = −0.584; *p* < 0.10) and TI (r = −0.567; *p* < 0.10). Starch was significantly positively correlated with TI (r = 0.668; *p* < 0.05) and ADF (r = 0.947; *p* < 0.01) contents. Glycinin was significantly positively correlated with β-conglycinin (r = 0.890; *p* < 0.01) and lectin (r = 0.886; *p* < 0.01) contents. The β-conglycinin was significantly positively correlated with lectin (r = 0.726; *p* < 0.05). The DE was significantly positively correlated with ME (r = 0.933; *p* < 0.01).

From FSBM chemical characteristics, the optimal regression equations for the DE and ME of FSBM were shown in Table 12. The best-fit equations were DE [MJ/kg DM] = 21.05 – 1.01 × CF + 0.04 × β-conglycinin (R^2^ = 0.61) and ME [MJ/kg DM] = 16.80 + 1.12 × DE – 1.03 × GE (R^2^ = 0.94).

### 3.4. The AID and SID of AA in FSBM

The averaged AID of Lys, Met, Trp, and Thr for FSBM samples was 72.55, 71.66, 74.84, and 71.05%, respectively (Table 13). The AID of Arg, Ala, Asp, Glu, and Gly for FSBM samples did not differ. The AID of Lys in FSBM sample 10 was the greatest, while the AID of Lys in FSBM samples 2 and 3 was the lowest (*p* < 0.01). The AID of Met in FSBM sample 7 was greater than in FSBM samples 2, 3, 4, 5, 6, and 9, and the AID of Met in FSBM sample 5 was the lowest (*p* < 0.01). The AID of Thr in FSBM sample 7 was the greatest, while the AID of Thr in FSBM samples 2 and 5 was the lowest (*p* = 0.02). The AID of Trp in FSBM sample 4 was the greatest, while the AID of Trp in FSBM samples 6 and 7 was the lowest (*p* < 0.01). There were no differences in the AID of Arg, Ala, Asp, Glu, and Gly (*p* > 0.05).

The averaged SID of Lys, Met, Trp, and Thr for FSBM samples was 76.62, 75.25, 80.96, and 77.90%, respectively (Table 14). The SID of Thr, Arg, Ala, Asp, Cys, Glu, Gly, and Ser for FSBM samples did not differ (*p* > 0.05). The SID of Lys in FSBM samples 7 and 10 was the greatest, while the SID of Lys in FSBM sample 3 was the lowest (*p* < 0.01). The SID of Met in FSBM sample 7 was greater than in FSBM samples 2, 3, 4, 5, 6, and 9, and the SID of Met in FSBM sample 5 was the lowest (*p* < 0.01). The SID of Trp in FSBM sample 4 was the greatest, while the SID of Trp in FSBM samples 6 and 7 was the lowest (*p* < 0.01). There were no differences in SID of Arg, Ala, Asp, Cys, Glu, Gly, and Ser (*p* > 0.05).

## 4. Discussion

All pigs remained healthy and they did not receive any preventive treatment during the experiment.

### 4.1. Chemical Composition of FSBM Samples

In this study, the GE value of the 10 FSBM samples ranged from 18.00 to 18.95 MJ/kg DM, with a mean of 18.44 MJ/kg DM, which is similar to the value reported by the NRC (2012) (GE: 18.97 MJ/kg DM) [1]. The CP content of the 10 FSBM samples in this study was comparable to that reported by Suprayogi et al. (2022) (50.16%) [25], lower than Kim et al. (2010) (55.3%) [26], and higher than Li et al. (2024) (46.00%) [27], falling within a reasonable range. The quality of FSBM may have been inconsistent because of differences in the soybean meal, strain used, and fermentation technology.

FSBM is fermented from soybean meal, so the nutrient level in soybean meal can influence the quality of FSBM (Chi and Cho, 2016) [28]. The EE and fiber content of FSBM samples from various sources in this study displayed significant variations. The chemical composition differences among FSBM from different sources are associated with the source of soybeans and the processing technology used in FSBM production (Chen et al., 2010; Yuan et al., 2017) [5,29]. Wang et al. (2022) analyzed the chemical composition of 11 soybean meal samples processed from different countries and found a coefficient of variation (CV) greater than 21.92% in EE, CF, ADF, and NDF contents [30]. This indicates that the choice of soybean meal significantly impacts the quality of FSBM.

The presence of soybean hulls, and whether the soybeans are peeled before processing, can greatly affect the CF content in FSBM (Lee et al., 2021) [31]. The selection of pressing technology and parameters during soybean meal production, such as temperature, humidity, and residence time, can lead to substantial changes in residual EE content (Grieshop et al., 2003) [32], potentially accounting for variations in EE content in FSBM.

Researchers fermented soybean meal with single bacteria, double bacteria mix, and triple bacteria mix and added different proportions of bacteria. It was found that CP, ash, Ca, and P contents were different among different bacteria, and multi-bacteria fermentation was better than single bacteria fermentation (Postigo et al., 2021) [33]. Upadhaya and Kim (2015) showed that different strains had different efficiency in improving soybean meal protein, *Bacillus subtilis* (*B. subtilis*) increased CP by 13.5%, while fermentation with *E. faecium* increased the CP by 10% [34]. Chi and Cho (2016) showed that *yeast* and *Bacillus subtilis* strains were more effective at increasing protein content compared to *lactic acid bacteria* (*LAB*). *Yeast* and *B. subtilis* strains convert carbohydrates in soybean meal into carbon dioxide gas, thereby increasing the relative protein content. In contrast, *LAB* tend to convert carbohydrates into organic acids, which contribute little to the improvement of protein content [28]. Chen et al. (2010) compared the effects of two-stage fermentation and single-stage fermentation on the quality of FSBM and proved that two-stage fermentation could increase the nutritional value as compared with single-stage fermentation [5].

Compared with soybean meal, the contents of soybean globulin (26.98 vs. 150.22 mg/g), β-conglycinin (36.13 vs. 123.20 mg/g), and trypsin inhibitor (0.33 vs. 11.16%) in FSBM were lower (Zhu et al., 2017) [35]. In this study, the content of anti-nutritional factors in FSBM was lower than that in soybean meal.

### 4.2. Energy Content of FSBM Samples

In this study, the means of the DE (17.55 MJ/kg DM) and ME (16.33 MJ/kg DM) of FSBM from 10 different sources were compared with the values reported by the NRC (2012) (DE: 17.91 MJ/kg DM; ME: 16.25 MJ/kg DM) [1], Rojas and Stein (2013) (DE: 17.97 MJ/kg DM; ME: 15.82 MJ/kg DM) [36], and Wang et al. (2014) (DE: 17.12 MJ/kg DM; ME: 16.54 MJ/kg DM) [37], demonstrating similarities. However, the ME values of the 10 FSBM samples exhibited a wide range from 14.30 to 18.05 MJ/kg DM. It can be seen that the database underestimates the DE and ME values of partial FSBM, leading to economic losses.

The effect of fermentation on the nutrient digestibility of soybean meal was also different. Min et al. (2009) found that FSBM could increase the ATTD of DM and CP in weaning pigs [38]. Yan and Kim (2013) reported that an FSBM diet had greater ATTD of DM, CP, and GE in growing pigs compared with an SBM diet [39]. FSBM is beneficial to the development of the gastrointestinal tract and improves the secretion of digestive enzymes, thereby improving the digestibility of nutrients (Kiarie et al., 2020) [40]. Wang et al. (2014) showed that there was no difference in DE and ME between FSBM and soybean meal [37]. Conversely, some studies have also shown that the fermentation of soybean meal will have a negative impact on the energy utilization of soybean meal (Rojasa and Stein, 2013; Jeong and Kim, 2015; Lan and Kim, 2020) [36,41,42].

Different strains of fermentation will also affect the digestibility of nutrients, and studies have shown that the digestibility of *Bacillus* is higher than that of a certain bacterium. FSBM affects the gut microbiome composition and thus the energy contribution of the hindgut microbiome (Muniyappan et al., 2023) [43]. Du et al. (2023) showed that the contribution rate of the hind intestine of growing pigs to energy is as high as 20% [44].

### 4.3. Correlations and Prediction Equations

Our results showed that there was a significant correlation between DE and ME, similar to previous studies (Le Goff and Noblet, 2001; Cozannet et al., 2010) [45,46]. Furthermore, we observed that CF had a differential tendency to be negatively correlated with DE and ME, which was also similar to previous studies (Li et al., 2015; Wang et al., 2022) [21,30]. Through stepwise regression, we identified CF as a predictor of DE (Wang et al., 2022) [30], but the R^2^ of the DE prediction equation in this experiment was relatively low. DE and GE can better predict ME (Zhuo et al., 2023) [47].

Although prediction equations for DE and ME in FSBM for growing pigs have been established in this study, a large amount of data is still required for continuous improvement, ultimately leading to a more accurate prediction equation model.

### 4.4. Digestibility of Amino Acids

The average SID of Lys (76.62%) and Trp (80.96%) was similar to that of NRC (2012) (75% and 78%) [1]. The SID of Met (75.25%) was lower than that of NRC (2012) (88%) [1], while the SID of Thr (77.90%) was higher than that of NRC (2012) (73%) [1]. The SID of the AA of the 10 FSBM samples in this study was lower than that reported by Wang et al. (2014) [37].

There are many factors affecting the SID of AA. The first is endogenous loss, and there are many methods to estimate the basic endogenous AA loss. The most commonly used method in growing pigs is the N-free diet method. Studies have shown that the use of N-free diets not only underestimates the basal endogenous nitrogen loss of pigs (Stein et al., 2007) [12], but also increases the basal endogenous Pro and Gly content (Moughan and Schuttert, 1991) [48]. In this study, the basal endogenous losses of Ala, Asp, Gly, and Glu were significant. Nyachoti et al. (1997) found that basic endogenous N loss varied between 1.8–8.3 g/kg DMI when a N-free diet was used [49]. Therefore, basic endogenous loss can significantly affect the accurate assessment of AA digestibility.

The other factor is the fermentation process. When soybean meal is fermented with different strains, the AA digestibility of the final product is different. Kim et al. (2015) found that FSBM fermented by *Bacillus subtilis* has a higher AID and SID of AA than FSBM fermented by *Aspergillus oryzae* [50]. Upadhaya and Kim (2015) reported that the fermentation of soybean meal by *Bacillus* shows a better digestibility of AA and nutrients [51]. Upadhaya et al. (2015) showed that protein solubility also affects the AA digestibility of FSBM [34]. In order to maintain the metabolic activity of microorganisms, the water content in the fermentation process of feed is high, and the fermented feed will be dried for preservation during production. The drying temperature will affect the digestibility of feed nutrients. Pahm et al. (2008) reported that heat losses caused by the Maillard reaction reduce AA digestibility [52]. The AA digestibility of FSBM sample 5 was lower and its color was brown. The increasing degree of Maillard reaction will lead to the aggravation of brown feed color (Murata, 2021; Sun et al., 2021) [53,54], which will reduce the nutrient digestibility (Salazar-Villanea et al., 2018) [55].

## 5. Conclusions

The chemical composition of FSBM in the Chinese market selected in this study has great variation, mainly reflected in fiber, mineral elements, and anti-nutritional factors. The average DE and ME of 10 FSBM samples were 17.55 and 16.33 MJ/kg DM, respectively, the average SID of Lys, Met, Trp, and Thr were 76.62%, 75.25%, 80.96%, and 77.90%, respectively. The DE, ME, and SID of the AA of FSBM from different sources varied greatly, and the chemical composition of FSBM can be used to predict DE and ME values.

## Figures and Tables

**Table 1:** Origins of fermented soybean meal (FSBM) samples.

No.	Facility ^1^	Location of Facility in China
1	A	Tianjin
2	B	Guangdong
3	C	Zhejiang
4	D	Hubei
5	E	Shanghai
6	F	Jiangsu
7	G	Fujian
8	G	Fujian
9	H	Shandong
10	I	Guangdong

^1^ The same capital letter means that the fermented soybean meal (FSBM) was processed in the same facility.

**Table 2:** Chemical composition of fermented soybean meal (FSBM) samples (as-fed basis, %) ^1^.

Items	FSBM No.										Mean	CV
	1	2	3	4	5	6	7	8	9	10		
DM ^2^	94.55	93.08	89.24	89.82	93.40	91.49	91.35	90.73	91.41	93.19	91.83	1.75
GE	18.95	18.47	18.00	18.13	18.76	18.35	18.51	18.47	18.29	18.51	18.44	1.52
CP	50.00	50.68	50.43	50.10	50.91	50.25	51.09	51.81	50.05	50.94	50.63	1.15
EE	1.75	0.69	1.23	1.14	0.86	1.12	1.17	1.62	0.93	1.08	1.16	27.59
Ash	6.27	6.83	6.14	6.42	6.17	6.15	5.92	6.24	6.25	6.34	6.27	3.83
NFE	31.80	30.42	27.00	27.92	30.36	30.17	27.77	27.65	28.84	30.69	29.26	5.57
CF	4.73	4.46	4.44	4.24	5.10	3.80	5.40	3.41	5.34	4.14	4.51	14.41
NDF	9.54	9.62	10.3	9.53	9.46	7.02	10.12	7.52	9.92	8.64	9.17	12.00
ADF	6.7	6.35	6.74	6.03	7.45	5.18	7.02	4.97	7.33	5.99	6.38	13.17
Starch	1.36	2.50	1.46	1.37	0.49	1.16	0.78	0.94	1.39	2.36	1.38	45.65
Phytic acid	0.18	0.29	0.28	0.24	0.28	0.25	0.23	0.24	0.24	0.23	0.25	12.00
Glycinin (mg/g)	73.00	50.19	59.77	45.19	61.25	30.79	70.69	65.54	46.00	75.00	57.74	24.85
Conglycinin (mg/g)	33.22	7.81	29.83	12.61	34.10	3.44	28.4	23.75	8.67	30.95	21.28	55.69
Trypsin inhibitor (mg/g)	7.46	9.59	8.40	8.86	5.75	7.32	8.02	7.89	7.38	8.37	7.90	13.16
Essential AA												
Lys	2.84	2.88	2.90	2.94	2.87	3.04	3.00	3.08	3.00	3.07	2.96	2.70
Met	0.51	0.56	0.52	0.59	0.55	0.55	0.62	0.60	0.55	0.48	0.55	7.27
Thr	1.90	1.93	1.98	1.95	1.92	1.92	2.57	2.49	1.91	1.92	2.05	11.71
Trp	0.64	0.66	0.66	0.65	0.64	0.64	0.67	0.68	0.67	0.64	0.66	1.52
Ile	2.17	2.22	2.27	2.26	2.25	2.27	2.28	2.28	2.25	2.26	2.25	1.33
Leu	3.63	3.77	3.82	3.76	3.73	3.77	3.79	3.84	3.74	3.75	3.76	1.33
Val	2.26	2.32	2.33	2.32	2.31	2.34	2.40	2.39	2.31	2.33	2.33	1.72
Arg	3.10	3.36	3.26	3.33	3.01	3.25	3.20	3.30	3.20	3.26	3.23	3.10
His	1.21	1.24	1.23	1.21	1.20	1.22	1.22	1.24	1.21	1.23	1.22	0.82
Phe	2.43	2.49	2.56	2.53	2.51	2.48	2.48	2.51	2.48	2.51	2.50	1.20
Nonessential AA												
Ala	2.19	2.21	2.17	2.17	2.24	2.20	2.31	2.31	2.18	2.18	2.22	2.25
Asp	5.37	5.42	5.52	5.53	5.50	5.53	5.46	5.64	5.48	5.49	5.49	1.28
Cys	0.60	0.65	0.62	0.62	0.62	0.60	0.61	0.62	0.60	0.51	0.61	4.92
Glu	8.99	9.16	9.28	9.28	9.22	9.27	9.00	9.21	9.22	9.21	9.18	2.18
Gly	2.03	2.06	2.08	2.06	2.07	2.04	2.17	2.14	2.05	2.05	2.08	1.92
Pro	2.44	2.59	2.50	2.49	2.52	2.50	2.43	2.49	2.48	2.48	2.49	1.61
Ser	2.40	2.48	2.54	2.48	2.46	2.45	2.43	2.47	2.45	2.45	2.46	1.22
Tyr	1.56	1.64	1.62	1.65	1.61	1.63	1.62	1.64	1.58	1.57	1.61	1.86

^1^ Fermented soybean meal, sources are described in Table 1. ^2^ DM, Dry matter; GE, gross energy; CP, crude protein; EE, ether extract; CF, crude fiber; NDF, neutral detergent fiber; ADF, acid detergent fiber; NFE, nitrogen-free extract; CV, coefficient of variation.

**Table 3:** Ingredient composition of experimental diets in experiment 1 (as-fed basis, %).

Ingredients	BASAL DIET	FSBM Diets (*n* = 10) ^1^
Corn	97.00	67.90
Fermented soybean meal	-	29.10
Dicalcium phosphate	1.20	1.20
Limestone	1.00	1.00
Sodium chloride	0.30	0.30
Vitamin–mineral premix ^2^	0.50	0.50
Total	100.00	100.00

^1^ The addition ratio of the ten fermented soybean meal (FSBM) samples in the diet remained the same. ^2^ The premix provided the following per kilogram of diets: 1500 IU of vitamin A; 200 IU of vitamin D_3_; 10 IU of vitamin E; 0.5 mg of vitamin K_3_; 1.0 mg of vitamin B_1_; 2.5 mg of riboflavin; 1.0 mg of vitamin B_6_; 10.0 μg of vitamin B_12_; 10.0 mg of niacin; 10.0 mg of pantothenic acid; 0.3 mg of folic acid; 0.05 mg of biotin; 0.50 g of choline; 80 mg of iron; 10 mg of copper; 80 mg of zinc; 30 mg of manganese; 0.3 mg of iodine; and 0.30 mg of selenium.

**Table 4:** Chemical composition of the experimental diets in experiment 1 (as-fed basis, %).

Item	DM ^1^	GE, MJ/kg	CP	Ash	EE	CF	NDF	ADF	Ca	P
Basal diet	87.15	15.86	8.89	3.79	2.81	1.96	8.36	3.19	0.88	0.48
FSBM ^2^ diet										
1	89.45	16.40	20.84	5.26	2.35	2.61	8.79	3.9	0.92	0.58
2	88.94	16.43	21.32	5.48	2.2	2.7	8.68	3.97	0.95	0.63
3	87.84	16.34	21.21	5.32	2.33	2.76	8.97	4.13	0.95	0.59
4	87.94	16.30	20.66	5.08	2.23	2.45	8.39	3.94	0.90	0.58
5	89.05	16.52	21.20	5.08	2.13	2.85	8.78	4.3	0.94	0.58
6	88.52	16.35	20.89	5.12	2.36	2.35	8.16	3.73	0.96	0.59
7	88.49	16.44	21.39	5.04	2.18	2.83	8.69	4.33	0.95	0.59
8	88.38	16.35	21.94	5.13	2.22	2.32	8.2	3.65	0.94	0.61
9	88.46	16.43	20.72	5.02	2.14	2.86	9.02	4.16	0.94	0.58
10	89.00	16.54	21.36	5.06	2.41	2.72	8.53	4.03	0.94	0.59

^1^ DM, Dry matter; GE, gross energy; CP, crude protein; EE, ether extract; CF, crude fiber; NDF, neutral detergent fiber; ADF, acid detergent fiber; Ca, calcium; P, phosphorus; CV, coefficient of variation. ^2^ FSBM, fermented soybean meal, sources are described in Table 1.

**Table 5:** Ingredient composition of experimental diets in experiment 2 (as-fed basis, %).

Ingredients	FSBM Diet (*n* = 10) ^2^	NF Diet ^3^
Fermented soybean meal	40.00	-
Cornstarch	44.45	79.55
Sucrose	10.00	10.00
Soybean oil	3.00	3.00
Cellulose	-	4.00
Dicalcium phosphate	1.10	1.40
Limestone	0.70	0.90
Sodium chloride	0.30	0.30
Potassium carbonate	-	0.30
Magnesium oxide	-	0.10
Titanium dioxide	0.20	0.20
Vitamin–mineral premix ^1^	0.25	0.25
Total	100.00	100.00

^1^ Premix provided the following per kg of complete diet: 1500 IU of vitamin A; 200 IU of vitamin D_3_; 10 IU of vitamin E; 0.5 mg of vitamin K_3_; 1.0 mg of vitamin B_1_; 2.5 mg of riboflavin; 1.0 mg of vitamin B_6_; 10.0 μg of vitamin B_12_; 10.0 mg of niacin; 10.0 mg of pantothenic acid; 0.3 mg of folic acid; 0.05 mg of biotin; 80 mg of iron; 10 mg of copper; 80 mg of zinc; 30 mg of manganese; 0.3 mg of iodine; and 0.30 mg of selenium. ^2^ The addition ratio of the ten fermented soybean meal (FSBM) samples in the diet remained the same. ^3^ NF = nitrogen-free.

**Table 6:** Amino acid (AA) composition of experimental diets in experiment 2 (as-fed basis, %).

Items	FSBM Diet Number ^1^									
	1	2	3	4	5	6	7	8	9	10
Dry matter	91.83	91.67	89.97	90.23	91.65	91.10	90.80	90.54	90.92	91.66
Essential AA										
Lys	1.09	0.96	0.94	1.09	1.03	1.09	1.12	1.17	1.03	1.16
Met	0.18	0.14	0.14	0.13	0.12	0.16	0.21	0.15	0.16	0.16
Thr	0.78	0.73	0.83	0.80	0.76	0.80	1.04	1.00	0.79	0.79
Trp	0.12	0.13	0.16	0.21	0.10	0.08	0.07	0.10	0.08	0.05
Ile	0.86	0.80	0.91	0.89	0.85	0.90	0.92	0.90	0.89	0.88
Leu	1.54	1.44	1.62	1.59	1.51	1.59	1.63	1.60	1.56	1.54
Val	0.91	0.86	0.97	0.95	0.91	0.97	1.02	1.00	0.97	0.95
Arg	1.23	1.17	1.19	1.27	1.14	1.29	1.31	1.29	1.25	1.27
His	0.49	0.45	0.48	0.48	0.46	0.49	0.49	0.49	0.47	0.49
Phe	1.00	0.93	1.07	1.03	0.99	1.03	1.04	1.03	1.03	1.01
Nonessential AA										
Ala	0.92	0.85	0.93	0.91	0.91	0.94	1.00	0.98	0.92	0.91
Asp	2.21	2.05	2.32	2.28	2.20	2.31	2.34	2.33	2.26	2.25
Cys	0.18	0.15	0.18	0.17	0.16	0.21	0.21	0.20	0.21	0.20
Glu	3.55	3.32	3.72	3.66	3.50	3.68	3.68	3.65	3.65	3.59
Gly	0.85	0.79	0.88	0.85	0.83	0.86	0.93	0.89	0.85	0.85
Ser	1.00	0.94	1.05	1.02	0.98	1.03	1.04	1.02	1.01	1.01
Tyr	0.58	0.47	0.55	0.54	0.52	0.60	0.64	0.59	0.59	0.54
Trp	0.12	0.13	0.16	0.21	0.10	0.08	0.07	0.10	0.08	0.05
Total AA	18.46	17.14	18.94	18.87	17.96	19.07	19.73	19.40	18.71	18.65

^1^ FSBM = fermented soybean meal, sources of FSBM in each FSBM number diet are same in Table 1.

**Table 7:** Mineral content of fermented soybean meal samples (as-fed basis) ^1^.

No.	Ca (%)	P (%)	Cu (mg/kg)	Zn (mg/kg)	Fe (mg/kg)	Mn (mg/kg)	Na (mg/kg)	Mg (%)	K (g/kg)
1	0.33	0.72	14.90	54.30	184.25	44.52	92.00	0.34	25.00
2	0.33	0.84	10.15	61.08	200.35	34.97	150.00	0.40	26.00
3	0.33	0.67	12.54	53.74	195.27	38.85	410.00	0.37	24.00
4	0.34	0.67	13.58	51.01	226.58	40.25	480.00	0.36	25.00
5	0.30	0.62	12.69	47.57	135.26	30.84	52.00	0.35	25.00
6	0.29	0.61	12.88	45.22	144.83	33.00	280.00	0.32	23.00
7	0.29	0.63	12.46	43.55	173.63	37.75	220.00	0.32	24.00
8	0.34	0.68	12.70	43.23	163.02	38.33	160.00	0.32	24.00
9	0.30	0.61	12.90	45.07	246.99	33.24	190.00	0.34	24.00
10	0.31	0.63	12.22	47.26	190.53	33.79	44.00	0.34	25.00
Mean	0.32	0.67	12.70	49.20	186.07	36.55	212.55	0.35	24.50
Maximum	0.34	0.84	14.90	61.08	246.99	44.52	480.00	0.40	26.00
Minimum	0.29	0.61	10.15	43.23	135.26	30.84	44.00	0.32	23.00
CV	6.25	10.48	8.82	11.10	17.53	10.73	70.07	8.57	3.47

^1^ Fermented soybean meal, sources are described in Table 1. CV, coefficient of variation.

**Table 8:** Anti-nutritional factors in fermented soybean meal samples (as-fed basis) ^1^.

No.	Phytic Acid (%)	Glycinin (mg/g)	β-Conglycinin (mg/g)	Trypsin Inhibitor (mg/g)	Lectin (mg/g)
1	0.18	73.00	33.22	7.46	6.67
2	0.29	50.19	7.81	9.59	4.35
3	0.28	59.77	29.83	8.40	5.15
4	0.24	45.19	12.61	8.86	4.48
5	0.28	61.25	34.10	5.75	3.46
6	0.25	30.79	3.44	7.32	0.37
7	0.23	70.69	28.40	8.02	5.68
8	0.24	65.54	23.75	7.89	4.89
9	0.24	46.00	8.67	7.38	1.79
10	0.23	75.00	30.95	8.37	7.63
Mean	0.25	57.74	21.28	7.90	4.45
Maximum	0.29	75.00	34.10	9.59	7.63
Minimum	0.18	30.79	3.44	5.75	0.37
CV	12.00	24.85	55.69	13.16	48.54

^1^ Fermented soybean meal, sources are described in Table 1. CV, coefficient of variation.

**Table 9:** DE and ME in basal and 10 fermented soybean meal experimental diets for growing pigs (dry matter basis) ^1^.

Item	DM	DE (MJ/kg)	ME (MJ/kg)	ME/DE (%)
Basal diets	87.15	16.12	15.72	97.54
No.				
1	94.55	15.88	15.18	95.55
2	93.08	15.66	15.01	95.85
3	89.24	16.36	15.81	96.67
4	89.82	16.00	15.39	96.23
5	93.40	15.69	14.97	95.42
6	91.49	15.98	15.46	96.69
7	91.35	15.87	15.37	96.84
8	90.73	16.10	15.42	95.75
9	91.41	15.60	14.96	95.86
10	93.19	16.10	15.59	96.82
Mean	91.83	15.92	15.32	96.17
CV	1.84	1.47	1.84	0.57

^1^ Basal diets and 10 fermented soybean meal experimental diets, sources are described in Table 3. DE, digestible energy; ME, metabolizable energy; CV, coefficient of variation.

**Table 10:** DE and ME in 10 fermented soybean meal samples for growing pigs (dry matter basis) ^1^.

No.	DE (MJ/kg)	ME (MJ/kg)	ME/DE (%)
1	17.92	15.98 ^abc^	89.24 ^d^
2	17.03	15.58 ^bc^	91.42 ^bcd^
3	18.80	18.05 ^a^	96.03 ^a^
4	16.79	15.89 ^bc^	94.59 ^abc^
5	17.21	15.58 ^bc^	90.49 ^cd^
6	18.58	17.72 ^ab^	95.40 ^ab^
7	16.99	16.21 ^abc^	95.38 ^ab^
8	17.93	16.34 ^abc^	91.05 ^bcd^
9	15.72	14.30 ^c^	90.71 ^cd^
10	18.50	17.68 ^ab^	95.54 ^ab^
Mean	17.55	16.33	92.99
SEM	0.70	0.75	1.54
*p*-value	0.07	0.03	0.01

^1^ Fermented soybean meal, sources are described in Table 1. DE, digestible energy; ME, metabolizable energy. ^a,b,c,d^ Means in the same column with different superscript letters are significantly different at *p* < 0.05.

**Table 11:** Correlation analysis of chemical composition, DE, and ME of fermented soybean meal (n = 10) (dry matter basis).

Item ^1^	DM	CP	EE	CF	NDF	ADF	Ash	GE	Starch	PA	Glycinin	β-Conglycinin	TI	Lectin	DE
CP	−0.043														
EE	−0.022	0.114													
CF	0.237	−0.312	−0.344												
NDF	−0.033	−0.326	−0.246	0.785 ***											
ADF	0.236	−0.320	−0.352	0.947 ***	0.854 ***										
Ash	0.261	−0.134	−0.377	−0.253	0.022	−0.166									
GE	0.901 ***	0.157	0.265	0.218	−0.090	0.189	−0.019								
Starch	0.203	−0.184	−0.298	−0.224	0.068	−0.172	0.755 **	−0.165							
PA	−0.466	0.115	−0.654 **	−0.073	0.100	0.051	0.209	−0.584 *	0.037						
Glycinin	0.296	0.489	0.443	0.165	0.296	0.226	−0.243	0.450	−0.019	−0.442					
β-conglycinin	0.235	0.360	0.420	0.206	0.307	0.338	−0.430	0.424	−0.273	−0.271	0.890 ***				
TI	−0.419	−0.021	−0.086	−0.314	0.160	−0.334	0.524	−0.567 *	0.668 **	0.190	−0.064	−0.334			
Lectin	0.208	0.307	0.393	−0.010	0.328	0.068	0.055	0.260	0.303	−0.405	0.886 ***	0.726 **	0.310		
DE	−0.022	0.195	0.403	−0.616 *	−0.449	−0.510	−0.181	−0.008	0.123	0.028	0.217	0.348	0.036	0.279	
ME	−0.246	0.151	0.236	−0.551 *	−0.368	−0.489	−0.253	−0.266	0.157	0.144	0.120	0.238	0.191	0.236	0.933 ***

^1^ DM, dry matter; GE, gross energy; CP, crude protein; EE, ether extract; CF, crude fiber; NDF, neutral detergent fiber; ADF, acid detergent fiber; PA, phytic acid; TI, trypsin inhibitor; DE, digestibility energy; ME, metabolizable energy; * *p* < 0.10, ** *p* < 0.05, *** *p* < 0.01.

**Table 12:** Linear regression equations for DE and ME based on chemical composition of fermented soybean meal (dry matter basis).

Item ^1^	Linear Regression Equations ^2^	R^2^	RMSE	*C(p)*	AIC	*p*-Value
1	DE (MJ/kg) = 21.50 − 1.01CF (%) + 0.04β-conglycinin (mg/g)	0.61	0.68	3.00	10.50	0.036
2	ME (MJ/kg) = 1.13DE (MJ/kg) − 3.43	0.87	0.45	2.00	13.60	<0.01
3	ME (MJ/kg) = 16.80 + 1.12DE (MJ/kg) − 1.03GE (MJ/kg)	0.94	0.33	3.00	8.70	<0.01

^1^ DE, digestible energy; CF, crude fiber; ME, metabolizable energy; GE, gross energy; RMSE, root mean square error is a measure of precision; C(*p*), conceptual predictive statistic; AIC, Akaike information criterion. ^2^ Regression equations were developed based on stepwise regression analyses.

**Table 13:** Apparent ileal amino acid digestibility of 10 fermented soybean meals samples (%).

Item	Number of Fermented Soybean Meal ^1^										Mean	SEM	*p*-Value	IAA ^2^ g/kg
	1	2	3	4	5	6	7	8	9	10				
Essential AA														
Lys	73.97 ^abc^	67.19 ^bc^	63.27 ^c^	72.14 ^abc^	69.74 ^abc^	71.07 ^abc^	78.33 ^ab^	75.69 ^ab^	71.87 ^abc^	79.24 ^a^	72.25	2.51	<0.01	5.09
Met	76.39 ^ab^	64.86 ^bc^	68.49 ^bc^	65.99 ^bc^	57.66 ^c^	69.19 ^bc^	86.73 ^a^	77.89 ^ab^	70.35 ^bc^	79.05 ^ab^	71.66	3.13	<0.01	0.60
Thr	72.37 ^ab^	66.86 ^b^	67.57 ^ab^	69.48 ^ab^	66.74 ^b^	69.53 ^ab^	79.03 ^a^	72.99 ^ab^	71.30 ^ab^	74.59 ^ab^	71.05	2.47	0.02	6.19
Trp	77.06 ^b^	80.79 ^ab^	81.61 ^ab^	87.55 ^a^	73.09 ^b^	64.86 ^c^	66.79 ^c^	74.29 ^b^	73.11 ^b^	69.20 ^b^	74.84	2.67	<0.01	0.63
Ile	81.99 ^ab^	78.24 ^ab^	73.70 ^b^	79.12 ^ab^	78.07 ^ab^	78.57 ^ab^	82.24 ^ab^	80.88 ^ab^	80.20 ^ab^	83.81 ^a^	79.68	1.83	0.03	4.36
Leu	83.99 ^a^	79.92 ^ab^	75.95 ^b^	80.88 ^ab^	80.92 ^ab^	81.66 ^ab^	84.50 ^a^	83.25 ^a^	82.19 ^ab^	84.29 ^a^	81.75	1.56	0.01	6.38
Val	78.82 ^ab^	74.33 ^ab^	71.51 ^b^	75.65 ^ab^	74.24 ^ab^	75.64 ^ab^	80.37 ^ab^	77.74 ^ab^	77.12 ^ab^	81.50 ^a^	76.69	1.96	0.03	5.09
Arg	86.59	81.68	82.27	85.72	84.00	85.05	87.45	87.18	87.33	90.17	85.74	2.11	0.20	7.49
His	81.75 ^ab^	76.03 ^ab^	72.91 ^b^	76.67 ^ab^	73.65 ^b^	76.66 ^ab^	81.49 ^ab^	78.91 ^ab^	77.79 ^ab^	84.10 ^a^	78.00	2.10	<0.01	2.19
Phe	85.04 ^a^	80.43 ^a^	73.77 ^b^	80.51 ^a^	81.35 ^a^	82.01 ^a^	84.09 ^a^	83.78 ^a^	82.71 ^a^	86.09 ^a^	81.98	1.61	<0.01	3.48
Nonessential AA														
Ala	74.98	64.33	68.11	71.76	71.31	71.89	76.77	72.81	62.08	75.10	70.91	2.71	0.08	8.23
Asp	76.61	71.52	70.84	77.10	71.00	73.22	78.47	71.83	75.47	79.11	74.52	2.35	0.08	9.69
Cys	68.62 ^a^	60.89 ^b^	56.37 ^c^	61.59 ^a^	57.73 ^b^	68.20 ^a^	71.16 ^a^	66.96 ^a^	66.07 ^a^	74.63 ^a^	65.22	5.36	0.04	1.20
Glu	78.32	70.31	68.69	73.50	71.61	72.01	78.59	74.60	81.05	80.46	74.91	2.90	0.09	12.90
Gly	63.54	58.08	54.10	59.30	52.34	56.14	66.43	59.94	48.95	68.66	58.75	3.74	0.06	21.04
Ser	79.85 ^ab^	73.47 ^c^	72.98 ^c^	77.57 ^abc^	75.55 ^bc^	77.14 ^abc^	81.44 ^a^	77.21 ^abc^	77.82 ^abc^	81.00 ^ab^	77.40	1.99	0.04	6.65
Tyr	80.30 ^a^	69.69 ^b^	67.80 ^b^	73.51 ^ab^	73.00 ^ab^	73.88 ^ab^	81.44 ^a^	77.27 ^ab^	75.36 ^ab^	81.39 ^a^	75.36	2.27	<0.01	2.95

^1^ Fermented soybean meal, sources are described in Table 1. ^2^ IAA: basic ileal endogenous loss of AA; IAA of Met (0.60 g/kg) was according to Yáñez et al. (2019) [24]. ^a,b,c,^ Means in the same row with different superscript letters are significantly different at *p* < 0.05.

**Table 14:** Standardized ileal amino acid digestibility of fermented soybean meals samples (%).

Item	Number of Fermented Soybean Meal ^1^										Mean	SEM	*p*-Value
	1	2	3	4	5	6	7	8	9	10			
Essential AA													
Lys	78.27 ^ab^	72.06 ^ab^	68.13 ^b^	76.36 ^ab^	74.28 ^ab^	75.31 ^ab^	82.47 ^a^	79.63 ^ab^	76.39 ^ab^	83.27 ^a^	76.62	2.51	<0.01
Met	79.4 ^ab^	68.79 ^bc^	72.32 ^bc^	70.16 ^bc^	62.23 ^c^	72.52 ^bc^	89.30 ^a^	81.51 ^ab^	73.82 ^bc^	82.43 ^ab^	75.25	3.13	<0.01
Thr	79.65	74.63	74.30	76.46	74.17	76.54	84.41	78.59	78.42	81.79	77.90	2.47	0.08
Trp	81.80 ^abc^	85.28 ^ab^	85.34 ^ab^	90.29 ^a^	78.88 ^abc^	72.30 ^c^	74.78 ^bc^	80.18 ^abc^	80.13 ^abc^	80.61 ^abc^	80.96	2.67	<0.01
Ile	86.65 ^a^	83.24 ^ab^	78.03 ^b^	83.54 ^ab^	82.75 ^ab^	82.97 ^ab^	86.53 ^ab^	85.27 ^ab^	84.63 ^ab^	88.34 ^a^	84.20	1.83	0.03
Leu	87.79 ^a^	83.98 ^ab^	79.50 ^b^	84.50 ^ab^	84.81 ^ab^	85.32 ^ab^	88.05 ^a^	86.86 ^a^	85.91 ^ab^	88.08 ^a^	85.48	1.56	0.01
Val	83.96 ^ab^	79.76 ^ab^	76.22 ^b^	80.49 ^ab^	79.37 ^ab^	80.45 ^ab^	84.92 ^ab^	82.35 ^ab^	81.91 ^ab^	86.41 ^a^	81.58	1.96	0.03
Arg	95.13	87.56	87.95	91.05	90.04	90.36	92.66	92.44	91.11	95.60	91.39	1.89	0.07
His	85.85 ^ab^	80.49 ^ab^	76.97 ^b^	80.78 ^ab^	78.01 ^b^	80.77 ^ab^	85.54 ^ab^	82.95 ^ab^	82.00 ^ab^	88.18 ^a^	82.15	2.10	0.01
Phe	88.23 ^a^	83.86 ^ab^	76.70^b^	83.56 ^ab^	84.58 ^a^	85.08 ^a^	87.12 ^a^	86.84 ^a^	85.79 ^a^	89.23 ^a^	85.10	1.61	<0.01
Nonessential AA													
Ala	83.19	73.20	76.08	79.92	79.60	79.88	84.25	80.42	82.62	83.42	80.26	2.71	0.13
Asp	80.63	75.85	74.60	80.94	75.04	77.05	82.22	75.60	79.37	83.06	78.44	2.35	0.09
Cys	74.72	63.98	62.38	67.94	64.43	73.34	77.59	72.37	71.36	81.08	70.92	5.36	0.09
Glu	81.65	73.87	71.81	76.68	74.99	75.20	81.78	77.80	75.33	83.75	77.29	2.90	0.09
Gly	86.27	82.49	75.53	81.63	75.46	78.48	87.06	81.34	81.30	91.45	82.10	3.74	0.08
Ser	85.96	79.96	78.69	83.45	81.76	83.04	87.22	83.11	83.80	87.07	83.41	1.99	0.06
Tyr	84.97 ^a^	75.44 ^ab^	72.66 ^b^	78.44 ^ab^	78.19 ^ab^	78.33 ^ab^	85.64 ^a^	81.79 ^ab^	79.9 1^ab^	86.38 ^a^	80.17	2.27	<0.01

^1^ Fermented soybean meal, sources are described in Table 1. ^a,b,c,^ Means in the same row with different superscript letters are significantly different at *p* < 0.05.

## Data Availability

The original contributions presented in the study are included in the article. Further inquiries can be directed to the corresponding author/s.

## References

[1] National Research Council, Division on Earth and Life Studies, Board on Agriculture and Natural Resources, Committee on Nutrient Requirements of Swine (2012). Nutrient Requirements of Swine.

[2] Woyengo T., Beltranena E., Zijlstra R. (2017). Effect of anti-nutritional factors of oilseed co-products on feed intake of pigs and poultry. Anim. Feed Sci. Technol..

[3] Zentek J., Boroojeni F.G. (2020). Technology. (Bio) Technological processing of poultry and pig feed: Impact on the composition, digestibility, anti-nutritional factors and hygiene. Anim. Feed. Sci. Technol..

[4] Zhang H., Yi J., Piao X., Li P., Zeng Z., Wang D., Liu L., Wang G., Han X.J. (2013). The metabolizable energy value, standardized ileal digestibility of amino acids in soybean meal, soy protein concentrate and fermented soybean meal, and the application of these products in early-weaned piglets. Asian-Australas. J. Anim. Sci..

[5] Chen C., Shih Y., Chiou P., Yu B.J. (2010). Evaluating nutritional quality of single stage-and two stage-fermented soybean meal. Asian-Australasian J. Anim. Sci..

[6] Jeong J.S., Park J.W., Lee S.I., Kim I.H.J. (2016). Apparent ileal digestibility of nutrients and amino acids in soybean meal, fish meal, spray-dried plasma protein and fermented soybean meal to weaned pigs. Anim. Sci. J..

[7] Li J., Zhou R.-l., Ren Z.-Q., Fan Y.-W., Hu S.-B., Zhuo C.-F., Deng Z.-Y. (2019). Improvement of protein quality and degradation of allergen in soybean meal fermented by Neurospora crassa. LWT-Food Sci. Technol..

[8] Yan H., Jin J., Yang P., Yu B., He J., Mao X., Yu J., Chen D.J. (2022). Fermented soybean meal increases nutrient digestibility via the improvement of intestinal function, anti-oxidative capacity and immune function of weaned pigs. Animal.

[9] Adeola O. (2000). Digestion and balance techniques in pigs. Swine Nutrition.

[10] Pedersen C., Boersma M., Stein H.J. (2007). Energy and nutrient digestibility in NutriDense corn and other cereal grains fed to growing pigs. J. Anim. Sci..

[11] Lyu Z., Chen Y., Wang F., Liu L., Zhang S., Lai C.J. (2023). Net energy and its establishment of prediction equations for wheat bran in growing pigs. Anim. Biosci..

[12] Stein H.H., Sève B., Fuller M., Moughan P., De Lange C.J. (2007). Invited review: Amino acid bioavailability and digestibility in pig feed ingredients: Terminology and application. J. Anim. Sci..

[13] (2014). Determination of Moisture in Feedstuffs.

[14] (2018). Determination of Crude Protein in Feeds—Kjeldahl Method.

[15] (2007). Determination of Ash in Feedstuffs.

[16] (2006). Determination of Ether Extract in Feedstuffs.

[17] (2018). Determination of Starch in Feeds.

[18] (2018). Determination of Calcium in Feeds.

[19] (2018). Determination of Phosphorus in Feeds-Spectrophotometry.

[20] (2017). Determination of the Contents of Calcium, Copper, Iron, Magnesium, Manganese, Potassium and Zinc in Feeds—Method Using Atomic Absorption Spectrometry.

[21] Li Z., Wang X., Guo P., Liu L., Piao X., Stein H.H., Li D., Lai C. (2015). Prediction of digestible and metabolisable energy in soybean meals produced from soybeans of different origins fed to growing pigs. Arch. Anim. Nutr..

[22] (2016). Determination of Titanium Dioxide in Food.

[23] Kaps M., William R.L. (2017). Biostatistics for Animal Science.

[24] Yáñez J.L., Woyengo T.A., Jha R., Van Kempen T.A., Zijlstra R.T. (2019). Nutrient digestibility of soybean products in grower-finisher pigs. J. Anim. Sci..

[25] Suprayogi W.P.S., Ratriyanto A., Akhirini N., Hadi R.F., Setyono W., Irawan A. (2022). Changes in nutritional and antinutritional aspects of soybean meals by mechanical and solid-state fermentation treatments with Bacillus subtilis and Aspergillus oryzae. Bioresour. Technol. Rep..

[26] Kim S., Van Heugten E., Ji F., Lee C., Mateo R. (2010). Fermented soybean meal as a vegetable protein source for nursery pigs: I. Effects on growth performance of nursery pigs. J. Anim. Sci..

[27] Li G., Wang Y., Zhang J., Xu S., Lin Y., Hua L., Li J., Feng B., Fang Z., Jiang X. (2024). Standardized ileal digestibility of amino acids in Chinese fermented soybean meal from different sources fed to mid and late-gestating sows. J. Anim. Sci..

[28] Chi C.-H., Cho S.-J. (2016). Improvement of bioactivity of soybean meal by solid-state fermentation with Bacillus amyloliquefaciens versus Lactobacillus spp. and Saccharomyces cerevisiae. LWT.

[29] Yuan L., Chang J., Yin Q., Lu M., Di Y., Wang P., Wang Z., Wang E., Lu F. (2017). Fermented soybean meal improves the growth performance, nutrient digestibility, and microbial flora in piglets. Anim. Nutr..

[30] Wang K., Zou X., Guo L., Huang L., Wang Y., Yang P., Huang L., Ma X., Zhuo Y., Che L. (2022). The nutritive value of soybean meal from different sources for sows during mid-and late gestation. J. Anim. Sci..

[31] Lee S.A., Park C.S., Kim B.G. (2021). Novel two-slope equations to predict amino acid concentrations using crude protein concentration in soybean meal. Agriculture.

[32] Grieshop C.M., Kadzere C.T., Clapper G.M., Flickinger E.A., Bauer L.L., Frazier R.L., Fahey G.C. (2003). Chemical and nutritional characteristics of United States soybeans and soybean meals. J. Agric. Food Chem..

[33] Postigo L.O.C.Y., Jacobo-Velázquez D.A., Guajardo-Flores D., Amezquita L.E.G., García-Cayuela T. (2021). Solid-state fermentation for enhancing the nutraceutical content of agrifood by-products: Recent advances and its industrial feasibility. Food Biosci..

[34] Upadhaya S.D., Ryu J.-h., Kang K.-i., Cho S.-J., Kim I.H. (2015). Effect of fermentation of soybean meal with varying protein solubility on ileal digestibility of nutrients in growing pigs. Anim. Prod. Sci..

[35] Zhu J., Gao M., Zhang R., Sun Z., Wang C., Yang F., Huang T., Qu S., Zhao L., Li Y. (2017). Effects of soybean meal fermented by L. plantarum, B. subtilis and S. cerevisieae on growth, immune function and intestinal morphology in weaned piglets. Microb. Cell Factories.

[36] Rojas O., Stein H.-H. (2013). Concentration of digestible, metabolizable, and net energy and digestibility of energy and nutrients in fermented soybean meal, conventional soybean meal, and fish meal fed to weanling pigs. J. Anim. Sci..

[37] Wang Y., Lu W., Li D., Liu X., Wang H., Niu S., Piao X. (2014). Energy and ileal digestible amino acid concentrations for growing pigs and performance of weanling pigs fed fermented or conventional soybean meal. Asian-Australas. J. Anim. Sci..

[38] Min B., Cho J., Chen Y., Kim H., Yoo J., Lee C., Park B., Lee J., Kim I. (2009). Effects of fermented soy protein on growth performance and blood protein contents in nursery pigs. Asian-Australas. J. Anim. Sci..

[39] Yan L., Kim I. (2013). Effect of probiotics supplementation in diets with different nutrient densities on growth performance, nutrient digestibility, blood characteristics, faecal microbial population and faecal noxious gas content in growing pigs. J. Appl. Anim. Res..

[40] Kiarie E.G., Parenteau I.A., Zhu C., Ward N.E., Cowieson A.J. (2020). Digestibility of amino acids, energy, and minerals in roasted full-fat soybean and expelled-extruded soybean meal fed to growing pigs without or with multienzyme supplement containing fiber-degrading enzymes, protease, and phytase. J. Anim. Sci..

[41] Jeong J.S., Kim I.H. (2015). Comparative efficacy of up to 50% partial fish meal replacement with fermented soybean meal or enzymatically prepared soybean meal on growth performance, nutrient digestibility and fecal microflora in weaned pigs. Anim. Sci. J..

[42] Lan R., Kim I. (2020). Science. Enterococcus faecium supplementation in sows during gestation and lactation improves the performance of sucking piglets. Vet. Med. Sci..

[43] Muniyappan M., Shanmugam S., Park J.H., Han K., Kim I.H. (2023). Effects of fermented soybean meal supplementation on the growth performance and apparent total tract digestibility by modulating the gut microbiome of weaned piglets. Sci. Rep..

[44] Du Z., Gao L., Wang Y., Xie J., Zeng S., Zhao J., Sa R., Zhao F. (2023). A comparative study on in vitro and in vivo stomach–small intestinal and large intestinal digestion of plant protein meals in growing pigs. J. Anim. Sci..

[45] Le Goff G., Noblet J. (2001). Comparative total tract digestibility of dietary energy and nutrients in growing pigs and adult sows. J. Anim. Sci..

[46] Cozannet P., Primot Y., Gady C., Métayer J., Lessire M., Skiba F., Noblet J. (2010). Energy value of wheat distillers grains with solubles for growing pigs and adult sows. J. Anim. Sci..

[47] Zhuo Y., Zou X., Wang Y., Jiang X., Sun M., Xu S., Lin Y., Hua L., Li J., Feng B. (2023). Nutritional values of cottonseed meal from different sources fed to gestating and non-pregnant sows. J. Anim. Sci..

[48] Moughan P.J., Schuttert G.J. (1991). Composition of nitrogen-containing fractions in digesta from the distal ileum of pigs fed a protein-free diet. J. Nutr..

[49] Nyachoti C., Lange C.d., McBride B., Schulze H.J. (1997). Significance of endogenous gut nitrogen losses in the nutrition of growing pigs: A review. Can. J. Anim. Sci..

[50] Kim D.H., Heo P.S., Jang J.C., Jin S.S., Hong J.S., Kim Y.Y. (2015). Effect of different soybean meal type on ileal digestibility of amino acid in weaning pigs. J. Anim. Sci. Technol..

[51] Upadhaya S.D., Kim I.H.J. (2015). Ileal digestibility of nutrients and amino acids in unfermented, fermented soybean meal and canola meal for weaning pigs. Anim. Sci. J..

[52] Pahm A., Pedersen C., Hoehler D., Stein H. (2008). Factors affecting the variability in ileal amino acid digestibility in corn distillers dried grains with solubles fed to growing pigs. J. Anim. Sci..

[53] Murata M.J. (2021). Browning and pigmentation in food through the Maillard reaction. Glycoconj. J..

[54] Sun Y., Lin L., Zhang P. (2021). Color development kinetics of Maillard reactions. Ind. Eng. Chem. Res..

[55] Salazar-Villanea S., Butré C.I., Wierenga P.A., Bruininx E.M., Gruppen H., Hendriks W.H., van der Poel A.F. (2018). Apparent ileal digestibility of Maillard reaction products in growing pigs. PLoS ONE.

